# Past-Year Violence Victimization is Associated with Viral Load Failure Among HIV-Positive Adolescents and Young Adults

**DOI:** 10.1007/s10461-020-02958-3

**Published:** 2020-08-06

**Authors:** Katherine G. Merrill, Jacquelyn C. Campbell, Michele R. Decker, John McGready, Virginia M. Burke, Jonathan K. Mwansa, Sam Miti, Christiana Frimpong, Caitlin E. Kennedy, Julie A. Denison

**Affiliations:** 1 Department of International Health, Johns Hopkins Bloomberg School of Public Health, 615 N. Wolfe Street, Baltimore, MD 21205 USA; 2 Department of Community-Public Health, Johns Hopkins School of Nursing, Baltimore, MD USA; 3 Department of Population, Family & Reproductive Health, Johns Hopkins Bloomberg School of Public Health, Baltimore, MD USA; 4 Department of Biostatistics, Johns Hopkins Bloomberg School of Public Health, Baltimore, MD USA; 5 Arthur Davison Children’s Hospital, Ndola, Zambia; 6 Present Address: Department of Medicine, University of Illinois at Chicago, Chicago, IL USA

**Keywords:** Violence, HIV, Viral load, Adolescent, Young adult, Zambia

## Abstract

We examined the relationship between past-year violence victimization and viral load (VL) failure among consecutively-sampled male and female adolescents and young adults, aged 15–24, in four HIV clinics in Ndola, Zambia. Measures of past-year physical violence, psychological abuse, and forced sex were adapted from the ICAST-C and WHO Multi-Country Study. Using logistic regression, we derived associations between VL failure (≥ 1000 copies/mL) and: any victimization; cumulative victimization; and types and perpetrators of violence. Among 272 youth (59.2% female, 72.8% perinatally infected), 73.5% (n = 200) experienced past-year violence and 36.8% (n = 100) had VL failure. Higher odds of VL failure were observed for participants who reported high frequency of any violence versus no violence victimization (adjusted OR, aOR: 3.58; 95% CI 1.14–11.27), high frequency of psychological abuse versus no psychological abuse (aOR: 3.32; 95% CI 1.26–8.70), any versus no violence from a family member other than a parent/caregiver for physical violence (aOR: 2.18, 95% CI 1.05–4.54) and psychological abuse (aOR: 2.50; 95% CI 1.37–4.54), and any versus no physical violence from a friend/peer (aOR: 2.14, 95% CI 1.05–4.36). Past-year violence victimization was associated with VL failure when considering the frequency, type, and perpetrator of violence. Programs addressing violence among youth living with HIV may be critical to improving viral suppression and preventing onward transmission.

**Electronic supplementary material:**

The online version of this article (10.1007/s10461-020-02958-3) contains supplementary material, which is available to authorized users.

## Introduction

Despite significant progress made in HIV prevention, care, and treatment in the past decade, HIV remains a leading cause of death among adolescents and young adults, ages 15–24 years, in sub-Saharan Africa [1]. Compared to adults, these youth in the region demonstrate lower levels of antiretroviral therapy (ART) adherence and viral suppression [2]. A national survey in Zambia found that only 34.3% of young people living with HIV ages 15–24 years had achieved viral suppression, compared to 79.0% of older adults ages 45–59 years [3].

Violence is also a leading cause of death among youth [1], and levels of violence against youth are among the highest in sub-Saharan Africa compared to other regions [4, 5]. In Zambia, 43% of female and 34% of male adolescents ages 13–17 experience past-year physical, emotional, or sexual violence [6]. Among young women aged 20–24 years, over one-third experience past-year physical violence and one-tenth experience past-year sexual violence [7].

Researchers are increasingly recognizing violence as a barrier to ART adherence and viral suppression among people living with HIV (primarily women) [8, 9], including in sub-Saharan Africa [10]. Threats or acts of violence from a controlling intimate partner can directly affect a woman’s ability to access the clinic for ART or adhere to their medication [11]. Violence victimization is also associated with greater likelihood of psychological distress, depression, and alcohol use [12], which are barriers to adherence among adults [13] and may thus prevent viral suppression and exacerbate risk of onward transmission.

Despite the growing literature on violence victimization and ART adherence/viral suppression among adult women, only three studies were identified among youth in sub-Saharan Africa [14–16]. These studies found associations between ART non-adherence and: violence exposure at home in Malawi [16] and violence from multiple perpetrators in the Eastern Cape, South Africa [14], among both male and female adolescents; and physical or sexual intimate partner violence (IPV) against female adolescents in Soweto, South Africa [15]. The study from the Eastern Cape found that non-adherence to ART increased with exposure to multiple types of victimization [14], echoing results from a study among perinatally-infected adolescents in the U.S. which found associations between higher levels of violence exposure and both unsuppressed viral load (> 400 copies/mL) and a CD4 of less than 25% [17].

A detailed assessment of the association between multiple forms of violence and HIV outcomes among youth in sub-Saharan Africa is critical to developing a holistic understanding of this public health problem and developing appropriate prevention and response efforts. Beyond looking at any experience of violence, we must understand the *cumulative* effects of violence on HIV outcomes, since exposure to multiple forms of violence (versus a single form) is associated with greater negative health outcomes [18]. Investigations into the unique contributions of specific forms of violence—e.g., the type of victimization (physical violence, psychological abuse, or forced sex) and perpetrator of violence—on HIV outcomes are needed to shed light on whether approaches to HIV care should be tailored to the type or perpetrator of violence. A study among HIV-positive women in Zambia, for instance, found that experiences of sexual and emotional IPV had stronger associations with ART adherence than physical IPV [19]. Furthermore, the association between violence victimization and VL failure may differ based on a youth’s sex or age group, given that violence exposure has shown differential effects for male and female youth [17, 20] and since young adulthood encompasses multiple developmental stages [21]. Strengthening the literature in these areas is particularly important since youth are undergoing cognitive, psychosocial, emotional, and social changes [21]; hence, we cannot assume that associations observed among adult women apply to youth, especially males.

Using data from adolescents and young adults living with HIV in Ndola, Zambia, we examined associations between viral load (VL) failure and past-year exposure to violence, including any victimization, cumulative victimization (i.e. frequency of violence and polyvictimization), types of victimization (physical, psychological, sexual), and perpetrators of violence. In line with the existing literature [18, 22, 23], we hypothesized that we would observe stronger associations with VL failure among youth who experience any violence, a higher frequency of any violence, and multiple types of violence, compared to those who experience no violence. We also investigated whether we would observe stronger associations with VL failure depending on the type or perpetrator of violence. Finally, we examined the presence of statistical interaction to determine whether any associations observed would differ according to the youth’s sex or age group.

### Methods

### Theoretical Approach

In taking a holistic approach to our analyses, we considered exposure to violence across multiple contexts, drawing on Kaufman’s socio-ecological framework [24]. Researchers have advocated for the use of socio-ecological frameworks in studies of violence [25] and HIV [24, 26], both for developing a deeper understanding of these multi-faceted health issues and for designing appropriate interventions. We focused our analyses on the individual and interpersonal levels, and considered the interpersonal (e.g. homes), institutional (e.g. clinics/schools), and structural (e.g. Zambian law) levels in formulating our study implications.

### Sample and Procedures

Analyses used cross-sectional baseline data from Project YES! (Youth Engaging for Success), a randomized controlled trial among youth living with HIV attending four clinics in Ndola, Zambia [27, 28]. The trial compared an intervention and comparison group to assess the effects of a peer-mentoring intervention on youths’ VL suppression (< 1000 copies/mL), ART treatment adherence (gap of 48 or more consecutive hours), and internalized/self-stigma [28]. Youth were consecutively sampled if they were: (a) aged 15–24 years, (b) aware of their HIV status, (c) on ART for 6 months or more, (d) a speaker of English or Bemba, and (e) available for study activities over 18 months (detailed elsewhere [28]).

In accordance with Zambian law, written informed consent was obtained from all participants age 18 and older [29]. For minors (ages 15–17 years), parental/caregiver permission and participant assent were obtained [29]. Participants completed baseline surveys between December 2017 to May 2018 in English or Bemba during face-to-face interviews, using Magpi software on tablet computers. Participants who reported experiences of severe violence or suicidal ideation were referred to designated healthcare providers at each clinic, according to the study’s safety protocol. Participants underwent blood draws for HIV-1 RNA viral load testing using the Qiagene QiAmp viral RNA mini kit (QIAGEN, Germany). Study teams also collected participants’ ART start dates from their medical records.

### Measures

#### Viral Load

Youth with a VL test of ≥ 1000 copies of HIV-RNA/mL were categorized as having VL failure, in line with consolidated guidelines on HIV treatment and prevention from the Zambian Ministry of Health and the World Health Organization (WHO) [30, 31].

#### Violence Victimization

Violence victimization was measured using items from the International Society for the Prevention of Child Abuse and Neglect Screening Tool-Child Instrument (ICAST-C) [32] and the WHO Multi-Country Study on Women’s Health and Domestic Violence (WHO MCS) [33]. Items assessed past-year experiences of physical violence (7 items), psychological abuse (6 items), and sexual violence (4 items) (Supplement 1). Items measuring physical violence were distinguished by severity level (three items for moderate, four items for severe violence) [33]. The act’s frequency in the past year was queried (never, once, a few times, many times), and 12 possible perpetrator types could be selected: romantic partner, parent/caregiver, other family member, friend/peer, stranger, school staff member, employer, health care worker, neighbor, religious leader, military/police, or someone else the youth knows. Three items assessing sexual violence were removed since they lacked clarity on whether the act was consensual [34]. Measures were translated into Bemba and the full instrument piloted among youth in Ndola for appropriateness.

Any victimization: Youth were classified as having experienced *any victimization* if reporting one or more behavioral acts of past-year violence (physical violence, psychological abuse, or forced sex) versus no acts.

Frequency of any victimization: A continuous measure was generated to offer insight into the accumulation of harm [35]; the *frequency of any victimization* was assessed by summing frequency scores across the 14 measures of violence (score range: 0-no frequency to 42-high frequency).

Polyvictimization: A categorical variable was generated for *polyvictimization* by grouping youth according to their experience of zero, one, or two or more types of past-year violence (physical violence, psychological abuse, or forced sex).

Types of victimization: Three measures assessed the specific types of violence experienced. A *severity-times-frequency measure of physical violence* was generated by multiplying the severity level (moderate-1, severe-2) by the frequency (never-0, once-1, a few times-2, many times-3) for each of the seven items and summing the scores across items (score range: 0-no severity/frequency to 42-high severity-times-frequency). This approach was modeled on the severity-times-frequency measure developed for the Conflict Tactics Scale [36]. The *frequency of psychological abuse* was assessed by summing frequency scores across the six items (score range: 0-no frequency to 18-high frequency). *Forced sex* was assessed as a binary variable (any versus no reports), given the small sample reporting this act.

Perpetrators of violence: Binary variables were generated for both any versus no reported physical violence and any versus no psychological abuse from the following *perpetrators*: parent/caregiver, other family member, romantic partner, and friend/peer. We distinguished perpetrators by the type of violence—i.e. physical violence and psychological abuse—for a more nuanced look at these forms of victimization. Associations for the remaining perpetrator types or for any perpetrator of forced sex were not assessed due to sparse data.

#### Covariates

Covariates were considered if potentially associated with violence victimization and VL failure, and not on the causal pathway between the two. Socio-demographic characteristics included the youth’s age (categorized as 15–19 or 20–24 years), sex, completion of primary school (yes or no), and orphan hood status (none, single orphan, or double orphan). HIV measures included the self-reported mode of HIV acquisition (from parents, through sex, or another way/don’t know/refused) and length of time on ART (6 months to 3 years, 3 to 6 years, or 6 + years). Study clinic was also included as a covariate.

### Analysis

Descriptive analyses were performed to estimate the proportion reporting VL failure, past-year violence, and the covariates of interest. Chi-square tests were used to assess differences in proportions by VL failure for all variables. Categorical measures of violence were generated from continuous measures based on locally weighted scatterplot smoothing (lowess) plots of the association between the variable and VL failure. We used this approach to make the models more robust against violations of the linearity assumption. We also conducted exploratory analyses to assess the overlap between the forms of violence experienced.

We built six logistic regression models to obtain crude and adjusted odds ratios (ORs), 95% confidence intervals (CIs), and p values (Wald tests) for the association between VL failure and: any victimization (binary) (Model 1); the frequency of any victimization (categorical) (Model 2); polyvictimization (categorical) (Model 3); the types of victimization, including severity-times-frequency of physical violence (categorical), frequency of psychological abuse (categorical), and forced sex (binary) (Model 4); the perpetrator of physical violence (indicator variables for each type) (Model 5); and the perpetrator of psychological abuse (indicator variables for each type) (Model 6). In all models, the reference group for the violence variable(s) consisted of those who had not experienced the form of violence being assessed. When exploring associations for the types of victimization (Model 4) and perpetrators of violence (Models 5 and 6), we included all variables assessing the violence type/perpetrator in adjusted models, alongside covariates, to determine whether any particular violence variable would show a stronger association with VL failure than the others. Missing item values were imputed as the referent, including completion of primary school (n = 1, 0.3% of sample) and time on ART (n = 3, 1.1% of sample).

All covariates were deemed theoretically important and therefore considered as candidates for inclusion in the six adjusted models. For each model, backwards elimination was used, where covariates were retained in adjusted models if reaching a significance level of 0.10 or if the covariate substantially influenced the OR of the main association of interest (+/− 10%) upon removal. All adjusted models included the youth’s sex and age, considered a priori covariates, and the study clinic as a fixed effect to account for the lack of independence of observations. Potential collinearity between any pairs of variables was examined using variance inflation factors. Hosmer–Lemeshow goodness of fit tests were conducted to assess the fit each model to the data. The final candidate multivariate models were extended to include an interaction term between the violence variable(s) and the youth’s sex and age group (15–19 versus 20–24 years), respectively. In post-hoc analyses, we stratified estimates by sex. Analyses were conducted in Stata 14 [37].

### Ethics

Study procedures aligned with the WHO ethical and safety recommendations [38], including: using broad terms to describe the research to youths’ caregivers in case the caregiver was perpetrating violence; addressing ethical considerations for violence research in the study staff training; minimizing under-reporting by avoiding judgmental or stigmatizing interpretation of youths’ experiences; and establishing a safety protocol to support violence victims. Ethical approval was obtained from the Johns Hopkins Bloomberg School of Public Health Review Board and the Zambian ERES Converge ethics review board. The research was reviewed and approved by the Zambian Ministry of Health through the National Health Research Authority.

## Results

Of 272 youth included in analyses, about two-thirds were female (59.2%, n = 161) and a similar proportion were aged 15–19 years (63.6%, n = 173) (Table 1). Most were perinatally infected (72.8%, n = 198), a single or double orphan (73.2%, n = 199), and had been on ART for 6 + years (61.0%, n = 166). About 88% (n = 240) had completed primary school. Almost three-quarters (73.5%, n = 200) reported any past-year physical violence, psychological abuse, or forced sex. Over a third had VL failure (36.8%, n = 101).

**Table 1:** Violence variables and covariates for the association between past-year violence victimization and viral load failure among adolescents and young adults living with HIV in Ndola, Zambia (n = 272), stratified by viral load failure

	Total 272 (100%)	Viral load		
		No failure 172 (63.2%)	Failure 100 (36.8%)	p value
Any victimization				
No violence	72 (26.5%)	46 (26.7%)	26 (26.0%)	0.89
Any physical violence, psychological abuse, or forced sex	200 (73.5%)	126 (73.3%)	74 (74.0%)	
Frequency of any victimization				
No violence (scores of 0)	72 (26.5%)	46 (26.7%)	26 (26.0%)	0.21
Single act of violence (scores of 1)	31 (11.4%)	22 (12.8%)	9 (9.0%)	
Moderate frequency (scores of 2–11)	150 (55.2%)	96 (55.8%)	54 (54.0%)	
High frequency (scores of 12–42)	19 (7.0%)	8 (4.7%)	11 (11.0%)	
Polyvictimization				
No violence	72 (26.4%)	46 (26.7%)	26 (26.0%)	0.77
1 type of violence	93 (34.2%)	61 (35.5%)	32 (32.0%)	
2 or 3 types of violence	107 (39.3%)	65 (37.8%)	42 (42.0%)	
Type of victimization				
Severity-times-frequency of physical violence				
No physical violence (scores of 0)	144 (52.9%)	94 (54.7%)	50 (50.0%)	0.72
Single act of physical violence (scores of 1)	34 (12.5%)	21 (12.2%)	13 (13.0%)	
Moderate severity-times-frequency (scores of 2–7)	56 (20.6%)	36 (20.9%)	20 (20.0%)	
High severity-times-frequency (scores of 8–42)	38 (14.0%)	21 (12.2%)	17 (17.0%)	
Frequency of psychological abuse				
No psychological abuse (scores of 0)	96 (35.3%)	61 (35.5%)	35 (35.0%)	0.03
Single act of psychological abuse (scores of 1)	31 (11.4%)	21 (12.2%)	10 (10.0%)	
Moderate frequency (scores of 2–5)	111 (40.8%)	76 (44.2%)	35 (35.0%)	
High frequency (scores of 6–18)	34 (12.5%)	14 (8.1%)	20 (20.0%)	
Forced sex				
No forced sex	258 (94.9%)	164 (95.4%)	94 (94.0%)	0.63
Any forced sex	14 (5.2%)	8 (4.7%)	6 (6.0%)	
Perpetrator of violence				
Physical violence from a:				
Parent/caregiver	42 (15.4%)	26 (15.1%)	16 (16.0%)	0.84
Other family member	39 (14.3%)	18 (10.5%)	21 (21.0%)	0.08
Romantic partner	16 (5.9%)	12 (7.0%)	4 (4.0%)	0.31
Friend/peer	44 (16.2%)	20 (11.6%)	24 (24.0%)	0.008
Psychological abuse from a:				
Parent/caregiver	39 (14.3%)	29 (16.9%)	10 (10.0%)	0.12
Other family member	68 (25.0%)	34 (19.8%)	34 (34.0%)	0.009
Romantic partner	24 (8.8%)	16 (9.3%)	8 (8.0%)	0.72
Friend/peer	99 (36.4%)	60 (34.9%)	39 (39.0%)	0.50
Covariates				
Sex				
Male	111 (40.8%)	66 (38.4%)	45 (45.0%)	0.28
Female	161 (59.2%)	106 (61.6%)	55 (55.0%)	
Age				
15–19	173 (63.6%)	107 (62.2%)	66 (66.0%)	0.53
20–24	99 (36.4%)	65 (37.8%)	34 (34.0%)	
Primary school (n = 271)				
Completed	240 (88.2%)	151 (87.8%)	90 (90.0%)	0.58
Did not complete	32 (11.8%)	21 (12.2%)	10 (10.0%)	
Mode of HIV acquisition				
From parents	198 (72.8%)	123 (71.5%)	75 (75.0%)	0.10
Through sex	27 (9.9%)	22 (12.8%)	5 (5.0%)	
Another way/don't know/refused	47 (17.3%)	27 (15.7%)	20 (20.0%)	
Time on antiretroviral therapy (n = 269)				
6 months to < 3 years	62 (22.8%)	42 (24.4%)	20 (20.0%)	0.44
3 to < 6 years	44 (16.2%)	30 (17.4%)	14 (14.0%)	
6+ years	166 (61.0%)	100 (58.1%)	66 (66.0%)	
Orphanhood				
None	73 (26.8%)	49 (28.5%)	24 (24.0%)	0.68
Single orphan	112 (41.2%)	68 (39.5%)	44 (44.0%)	
Double orphan	87 (32.0%)	55 (32.0%)	32 (32.0%)	
Clinic				
1	144 (52.9%)	85 (49.4%)	59 (59.0%)	0.34
2	35 (12.9%)	26 (15.1%)	9 (9.0%)	
3	64 (23.5%)	43 (25.0%)	21 (21.0%)	
4	29 (10.7%)	18 (10.5%)	11 (11.0%)	

Percentages are column percentages. Frequency of any victimization scores were generated by summing each act’s frequency (never-0, once-1, a few times-2, many times-3) across 14 acts of violence. Severity-times-frequency of physical violence scores were generated by multiplying the act’s severity level (moderate-1, severe-2) by its frequency (never-0, once-1, a few times-2, many times-3), and summing across 7 acts of physical violence. Frequency of psychological abuse scores were generated by summing each act’s frequency (never-0, once-1, a few times-2, many times-3) across 6 acts of psychological abuse. Categories for violence scores were determined based on locally-weighted scatterplot smoothing (lowess) plots for the association between the variable and viral load failure. P values are chi-square tests

No evidence of an association was observed for any past-year violence victimization as a binary variable and VL failure (Model 1). The small proportion (7%, n = 19) reporting a high frequency of any past-year victimization (scores of 12–42) had 3.58 times the odds of VL failure compared to those reporting no past-year violence (95% CI 1.14–11.27, p < 0.05), after adjusting for covariates (Model 2). No evidence of an association was observed for past-year polyvictimization and VL failure (Model 3) (Table 2).

**Table 2:** Crude and adjusted associations between past-year violence victimization and viral load failure among adolescents and young adults living with HIV in Ndola, Zambia (n = 272)

	Crude odds ratio	95% CI	p value	Adjusted odds ratio^a^	95% CI	p value
Model 1: any violence victimization						
No violence (n = 72)	1			1		
Any physical, psychological, or forced sex (n = 200)	1.04	(0.59, 1.82)	0.89	1.09	(0.61, 1.95)	0.77
Model 2: frequency of any victimization^b^						
No violence (scores of 0) (n = 72)	1			1		
Single act of violence (scores of 1) (n = 31)	0.72	(0.29, 1.80)	0.49	0.70	(0.28, 1.79)	0.46
Moderate frequency (scores of 2–11) (n = 150)	0.99	(0.55, 1.78)	0.97	1.06	(0.58, 1.96)	0.84
High frequency (scores of 12–42) (n = 19)	2.43	(0.87, 6.81)	0.09	3.58	(1.14, 11.27)	0.03
Model 3: polyvictimization						
No violence (n = 72)	1			1		
1 type of violence (n = 93)	0.93	(0.49, 1.77)	0.82	0.98	(0.50, 1.90)	0.94
2 or 3 types of violence (n = 107)	1.14	(0.62, 2.12)	0.67	1.21	(0.63, 2.29)	0.57
Model 4: type of victimization						
Severity-times-frequency of physical violence^b^						
No physical violence (scores of 0) (n = 144)	1			1		
Single act of physical violence (scores of 1) (n = 34)	1.16	(0.54, 2.52)	0.70	1.08	(0.47, 2.47)	0.85
Moderate severity-times-frequency (scores of 2–7) (n = 56)	1.04	(0.55, 1.99)	0.90	0.93	(0.45, 1.93)	0.84
High severity-times-frequency (scores of 8–42) (n = 38)	1.52	(0.74, 3.14)	0.26	1.18	(0.49, 2.85)	0.71
Frequency of psychological abuse^b^						
No psychological abuse (scores of 0) (n = 96)	1			1		
Single act of psychological abuse (scores of 1) (n = 31)	0.83	(0.35, 1.96)	0.67	0.84	(0.34, 2.04)	0.70
Moderate frequency (scores of 2–5) (n = 111)	0.80	(0.45, 1.42)	0.46	0.81	(0.42, 1.57)	0.54
High frequency (scores of 6–18) (n = 34)	2.49	(1.12, 5.53)	0.03	3.32	(1.26, 8.70)	0.01
Forced sex						
No forced sex (n = 258)	1			1		
Any forced sex (n = 14)	1.31	(0.44, 3.88)	0.63	1.19	(0.35, 4.01)	0.78
Model 5: perpetrator of physical violence						
Any vs. none from a parent/caregiver (n = 42 vs. 230)	1.07	(0.54, 2.10)	0.85	0.95	(0.46, 1.96)	0.89
Any vs. none from another family member (n = 39 vs.233)	2.27	(1.15, 4.51)	0.02	2.18	(1.05, 4.54)	0.04
Any vs. none from a romantic partner (n = 16 vs. 256)	0.56	(0.17, 1.77)	0.32	0.77	(0.21, 2.78)	0.69
Any vs. none from a friend/peer (n = 44 vs. 228)	2.40	(1.25, 4.62)	0.01	2.14	(1.05, 4.36)	0.04
Model 6: Perpetrator of psychological abuse						
Any vs. none from a parent/caregiver (n = 39 vs. 233)	0.55	(0.25, 1.18)	0.12	0.48	(0.21, 1.09)	0.08
Any vs. none from another family member (n = 68 vs. 204)	2.09	(1.19, 3.67)	0.01	2.50	(1.37, 4.54)	0.003
Any vs. none from a romantic partner (n = 24 vs. 248)	0.85	(0.35, 2.06)	0.72	1.14	(0.42, 3.10)	0.80
Any vs. none from a friend/peer (n = 99 vs. 173)	1.19	(0.72, 1.99)	0.50	1.18	(0.68, 2.05)	0.55

^a^Adjusted for age, sex, and study clinic (a priori), and the following: mode of HIV acquisition (all models), time on ART treatment (all models), orphan hood (all models except Model 3). Models 4–6 adjusted for the other violence variables in addition to covariates. p values are Wald tests

^b^Frequency of any victimization scores were generated by summing each act’s frequency (never-0, once-1, a few times-2, many times-3) across 14 acts of violence. Severity-times-frequency of physical violence scores were generated by multiplying the act’s severity level (moderate-1, severe-2) by its frequency (never-0, once-1, a few times-2, many times-3), and summing across 7 acts of physical violence. Frequency of psychological abuse scores were generated by summing each act’s frequency (never-0, once-1, a few times-2, many times-3) across 6 acts of psychological abuse. Categories for violence scores were determined based on locally-weighted scatterplot smoothing (lowess) plots for the association between the variable and viral load failure

Examining the types of violence (Model 4, Table 2) revealed that the 12.5% of the sample (n = 34) reporting a high frequency of past-year psychological abuse (scores of 6–18) had 2.49 times the odds of VL failure compared to those not reporting past-year psychological abuse (95% CI 1.12–5.53, p < 0.05) in crude analyses. This association strengthened to an OR of 3.32 (95% CI 1.26–8.70, p < 0.01), after adjusting for physical violence, forced sex, and covariates. In examining the overlap in types of violence experienced, we found that among those reporting a high frequency of past-year psychological abuse, about two-thirds (64.7%, n = 22) also reported a high frequency of past-year physical violence and/or any forced sex (not pictured).

Regarding perpetrators of violence (Models 5 and 6, Table 2), significant associations were observed for both past-year physical violence (aOR: 2.18, 95% CI 1.05, 4.54, p < 0.05) and psychological abuse (aOR: 2.50, 95% CI 1.37, 4.54, p < 0.01) from a family member other than a parent or caregiver. Additionally, youth who reported past-year physical violence from a friend/peer, compared to those who had not, had 2.14 times the odds of VL failure (95% CI 1.05–4.36, p < 0.05) after adjusting for violence from other perpetrator groups and covariates. We did not find evidence for an association between VL failure and past-year physical violence or psychological abuse from a parent/caregiver or romantic partner.

No significant interaction by sex or age group was observed for any models. We did, however, observe qualitative differences in both ORs and 95% CIs when examining the results stratified by sex in post-hoc analyses (Supplement 2). The significant associations with VL failure for a high frequency of any past-year victimization, a high frequency of past-year psychological abuse, and any versus no past-year physical violence or psychological abuse from a family member other than a parent/caregiver were observed among male but not female youth in sex-stratified adjusted models. The significant association with VL failure for any versus no past-year physical violence from a friend/peer was observed among female but not male youth. Among female youth, we observed a significant adjusted association with VL failure for any versus no past-year physical violence from a romantic partner in adjusted models only (aOR: 2.28, 95% CI 1.03–5.04, p < 0.05).

## Discussion

We found that past-year violence victimization among adolescents and young adults living with HIV was associated with VL failure when considering the frequency, type, and perpetrator of violence. Importantly, while we found no associations for any violence as a binary variable, the small proportion of youth categorized as experiencing a high frequency of any violence victimization (7%) showed higher odds of VL failure. In South Africa, Cluver et al. found a similar pattern of increasing risk of ART non-adherence by additional violence exposure among 1060 adolescents (10–19 years old) [14]. These results—including the lack of evidence for single acts or moderate frequency of violence—support the growing recognition of the need to consider cumulative effects of multiple types of violence on health outcomes [18, 22, 23] and specifically HIV outcomes [14, 17].

Experiencing a high frequency of past-year psychological abuse was significantly associated with VL failure, independent of experiences of physical violence and forced sex, in our examination of the unique contributions of violence types. It may be that for the small proportion of youth categorized as experiencing a high frequency of psychological abuse (12.5%), their experiences manifest in part as enacted HIV-stigma. Measures of HIV stigma among youth often include acts of verbal or emotional mistreatment [39], and qualitative studies have shed light on enacted HIV-stigma as a key concern facing youth living with HIV in sub-Saharan Africa [40, 41]. The effects of psychological abuse on youth may be compounded by the developmental stage of adolescence, during which they develop the skills in managing their emotions, relating with others, and feeling self-confident [21]. This analysis did not assess whether the youth believed the psychological abuse they were experiencing was due to their HIV status. Further research is needed to understand the contexts in which psychological abuse occurs, the forms it takes, and how it affects HIV outcomes among youth.

Other key findings concern the perpetrators of violence. We found that past-year experiences of physical violence and psychological abuse from a family member other than a parent/caregiver, as well as physical violence from a friend or peer, were independently associated with VL failure. Associations were strongest for psychological abuse from a family member other than a parent/caregiver. Echoing our findings for the association between VL failure and high frequency of psychological abuse, these results underscore the critical need for a deeper exploration of the meanings, drivers, and consequences of psychological abuse among HIV-positive youth in sub-Saharan Africa. While the quality of family engagement is known to affect ART adherence among youth in the region [42–44], we need to understand which family members perpetrate violence and what is the nature of their relationship with the youth, including whether they live in the same household. Additionally, some research has explored experiences of bullying among adolescents living with HIV in Malawi [16] and South Africa [45, 46], but further insight into experiences of physical violence from friends and peers, and the extent to which such violence occurs in or around schools, could inform intervention strategies.

Unlike previous studies in sub-Saharan Africa [14, 15], we did not find any associations with VL failure for past-year violence from a romantic partner, which could reflect a lack of statistical power in our study given that so few youth reported this form of violence. Nor did we find associations with past-year physical violence or psychological abuse from a parent/caregiver, whereas the Cluver et al. study found associations for physical abuse [14]. Youth in our study may have considered these forms of violence as disciplinary practices and thus their HIV self-care was less impacted than violence from other groups [47]. We also did not find statistical differences in the association between past-year violence victimization and VL failure by youths’ sex, in contrast with findings from a study of perinatally-infected HIV-positive adolescents in the U.S. which found that recent indirect exposure to violence was related to unsuppressed VL in boys but not girls [17]. We did, however, observe some differences in adjusted analyses stratified by sex. Among males, a high frequency of any victimization, a high frequency of psychological abuse, and any versus no physical or sexual violence from a family member other than a parent/caregiver in the past year were significantly associated with VL failure. In contrast, females showed higher odds of VL failure if reporting any versus no past-year physical violence from a friend/peer or psychological abuse from a romantic partner. Given our relatively small sample size and the small proportions reporting these forms of violence, more research in this area is needed.

The implications of these data should be considered in light of the structural, institutional, and interpersonal levels of Kaufman’s socio-ecological framework [24]. At the structural level, the Zambian government has taken seriously the need to address gender-based violence (GBV). It passed one of the most comprehensive GBV acts on the continent, designed to protect victims, establish an anti-GBV committee and fund, create shelters for and offer counseling to victims, and facilitate the issuance of protection orders [48]. Combined with our study data, this act—which includes provisions for addressing “emotional, verbal and psychological abuse” alongside physical and sexual violence [48]—provides a strong foundation on which to build screening and response initiatives at the institutional level, namely, in HIV clinics where routine violence screening among adolescents and young adults does not currently occur. This act also provides policy support for ensuring that such clinics have sufficient resources to properly respond to disclosures of violence [49]. Although almost three-quarters of our sample experienced some form of past-year violence victimization, we found that the relatively small proportion who experience a high frequency of any victimization—and psychological abuse specifically—were the ones with higher odds of VL failure and may benefit most from targeted interventions. Our data highlight the need for HIV clinics to screen youth for psychological abuse in addition to physical and sexual violence and provide support services [50, 51] to reduce VL failure among this disparate population.

Our findings have further implications for addressing violence occurring in schools at the institutional level and in homes at the interpersonal level. While rigorous evaluation of school-based interventions in sub-Saharan Africa is still lacking [52], reductions in peer violence have been observed following delivery of a school-wide intervention in Uganda [53, 54]. Our findings on the association of peer-perpetrated physical violence suggest that further investment to address violence by peers, perhaps through school-based approaches, should be investigated as a way to potentially prevent poor virologic outcomes among youth living with HIV. Furthermore, intervention efforts to engage caregivers in the health of youth living with HIV are underway in the region [52] and could provide a useful platform to address violence from other family members.

Study limitations must be acknowledged. Our data were cross-sectional; hence, we are unable to draw conclusions about the temporal ordering of the violence victimization-VL association. Although incomplete ART adherence is a primary means through which victimization may influence VL, longitudinal studies could formally explore adherence as a mediator of the association and account for other possible mediators (e.g. depression, alcohol use). We did not measure the frequency of past-year violence from specific perpetrator groups since this would have required a much longer questionnaire, preventing a more nuanced understanding of our significant findings that family and friend/peer violence were associated with VL failure. Our relatively small sample size may have resulted in lower-than-desired precision and prevented us from formally testing for synergistic interactions across violence types [55], though we still considered the frequency, severity, and multiple types of past-year violence exposure. Finally, our population was consecutively sampled from HIV clinics and had been on ART for at least 6 months; our findings may not be generalizable to youth living with HIV who are not in care or on ART, since victims of violence are typically less likely to engage in HIV care than non-victims [56, 57].

## Conclusions

Addressing violence may be critical to improving virologic outcomes and preventing the spread of HIV among adolescents and young adults in sub-Saharan Africa. Policies and programs are needed to support youth living with HIV who experience violence, especially those experiencing high frequency of any violence and a high frequency of psychological abuse. Data on perpetrators and types of violence will strengthen and allow for targeted responses to youth who are at increased likelihood of VL failure. Researchers should use longitudinal studies and qualitative methods to further explore pathways between violence victimization and virologic outcomes among both male and female youth living with HIV.

## Electronic supplementary material

Below is the link to the electronic supplementary material.

Electronic supplementary material 1 (DOCX 37 kb)
