# Supplementary material for: Past-Year Violence Victimization is Associated with Viral Load Failure Among HIV-Positive Adolescents and Young Adults
Source: AIDS Behav. 2020 Aug 6;25(5):1373–83. doi: 10.1007/s10461-020-02958-3 (PMC8052241; doi:10.1007/s10461-020-02958-3)
Supplement: Supplementary file 1 — Electronic supplementary material 1 (DOCX 37 kb) [file 10461_2020_2958_MOESM1_ESM.docx]

**Past-year violence victimization is associated with viral load failure among HIV-positive adolescents and young adults**

Supplement 1. Measures of violence victimization, adapted from the International Society for the Prevention of Child Abuse and Neglect Screening Tool-Child Instrument (ICAST-C) and the World Health Organization Multi-Country Study on Women’s Health and Domestic Violence against Women

| **Physical violence: Has anyone^…** |
| --- |
| Moderate violence:   - Slapped you or thrown something at you that could hurt you? - Pushed or shoved you? - Twisted your ear or arm as punishment? |
| Severe violence:   - Hit you with a fist or with something else that could hurt you, such as a stick or a cane? - Kicked you, dragged you, or severely beaten you up? - Choked you or burnt you on purpose? - Threatened to use or actually used a sharp object or other weapon against you? |
| **Psychological abuse: Has anyone^…** |
| - Insulted you or made you feel bad about yourself? - Belittled or humiliated you in front of other people? - Threatened to leave or abandon you? - Locked you either inside or outside of the home? - Threatened to invoke harmful people, ghosts or evil spirits against you? - Referred to your skin color/ gender/ religion/ tribe/ or health problems you have in a hurtful way? |
| **Sexual violence: Has anyone^…** |
| - Physically forced you to have sexual intercourse when you did not want to? - *Not included:* Made you watch a sex video or look at sexual pictures?** - *Not included:* Made you look at their private parts or wanted to look at yours?** - *Not included:* Touched your private parts or wanted to look at yours?** |

^Timeframes of violence assessed include lifetime and past-year. **Removed during analysis stage given concerns that the non-consensual nature of the act was unclear.

Supplement 2. Crude and adjusted associations between past-year violence victimization and viral load failure among adolescents and young adults living with HIV in Ndola, Zambia, stratified by sex (n=272)
